# Supplementary material for: Aequorea’s secrets revealed: New fluorescent proteins with unique properties for bioimaging and biosensing
Source: PLoS Biol. 2020 Nov 2;18(11):e3000936. doi: 10.1371/journal.pbio.3000936 (PMC7660908; doi:10.1371/journal.pbio.3000936)

16S Tree. The 16S tree is inconclusive as to the phylogenetic position of both the transcriptomic 16S sequences and the reference-guided assembly 16S sequence. Several species are monophyletic in this tree and A. australis is in a large polytomy.

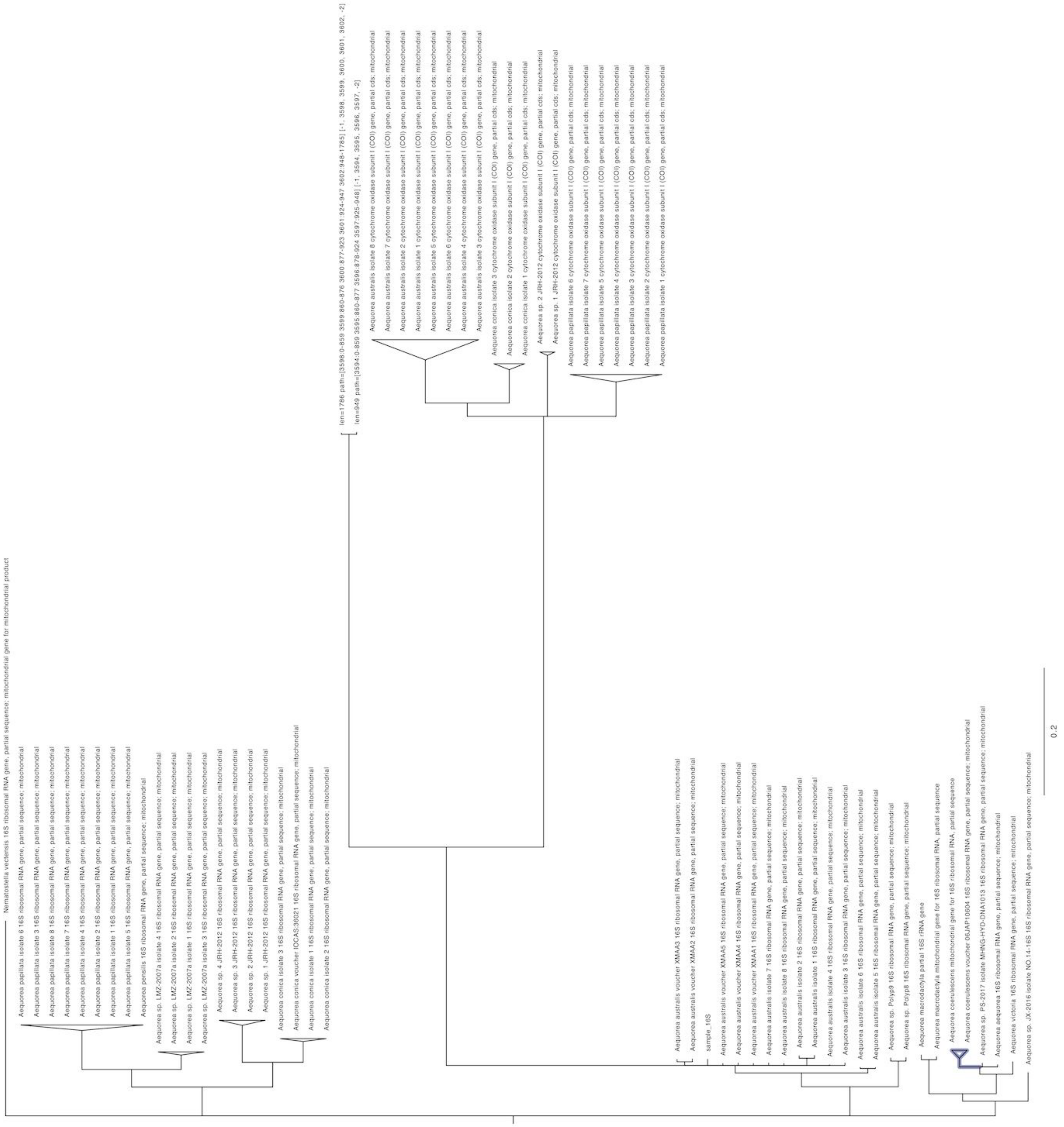

Supplement: S1 Fig — The 16S tree is inconclusive as to the phylogenetic position of both the transcriptomic 16S sequences and the reference-guided assembly 16S sequence. Several species are monophyletic in this tree and A. australis is in a large polytomy. See S1 Text for additional discussion. (PDF) [file pbio.3000936.s002.pdf]
