# Supplementary material for: Aequorea’s secrets revealed: New fluorescent proteins with unique properties for bioimaging and biosensing
Source: PLoS Biol. 2020 Nov 2;18(11):e3000936. doi: 10.1371/journal.pbio.3000936 (PMC7660908; doi:10.1371/journal.pbio.3000936)
Supplement: S2 Fig — The COI tree shows that the reference-corrected COI sequence (sample_COI) is sister to a large A. australis clade. See S1 Text for additional discussion. (PDF) [file pbio.3000936.s003.pdf]

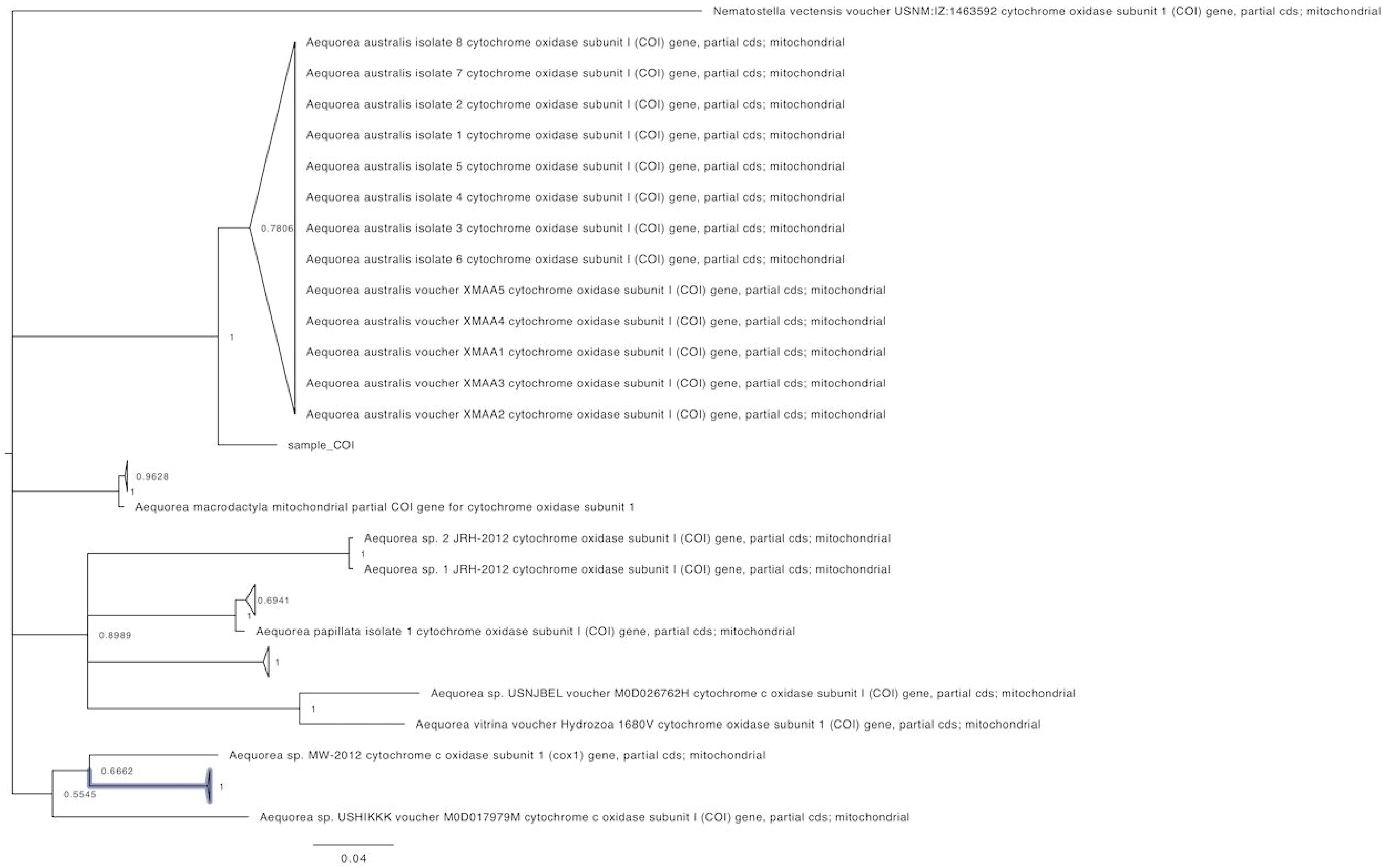

COI Tree. The COI tree shows that the reference-corrected COI sequence (sample_COI) is sister to a large A. australis clad. There was no COI sequence identified in the de novo transcriptome so there is no comparison. It is important to note that the sample's COI sequence is bona-fide, even though it was assembled using reference-guided assembly. Therefore the sequence coming out as sister to A. australis indicates true relation.
