# Supplementary material for: Aequorea’s secrets revealed: New fluorescent proteins with unique properties for bioimaging and biosensing
Source: PLoS Biol. 2020 Nov 2;18(11):e3000936. doi: 10.1371/journal.pbio.3000936 (PMC7660908; doi:10.1371/journal.pbio.3000936)
Supplement: Attachment — Submitted filename: Response to Reviewers PLoS Biology Revision 8-2020.pdf [file pbio.3000936.s007.pdf]

8-7-2020
Response to reviewers

We wish to thank the reviewers for their thoughtful and specific comments, which undoubtedly improved the manuscript and led to an even more thorough characterization of the novel FP homologs from *Aequorea* jellies. The following is a summary of the major changes made to the manuscript and supporting information, followed by a point-by-point response to the concerns, comments, and suggestions made by the reviewers.

Changes to figures and new figures

1. Figure 1 - the original figure 1 and panels A and C from figure 2 were combined into a single figure that captures the important information in the two original figures and eliminates redundant/irrelevant data.

2. Figure 2 - the original figure 3 was altered to increase the thickness of lines for visibility and to provide clear axis and scale labels, and to add arrows indicating the absorbance peaks that change with photoconversion or photoswitching.

3. Figure 3 - the original figure 4 (sequence alignment) was replaced with a simple phylogenetic tree illustrating the relationship between the proteins cloned in this study, as requested by reviewer 1.

4. Figure 4 - renumbered from original figure 5, unchanged.

5. Figure 5 - renumbered from original figure 6, unchanged.

6. Figure 6 - renumbered from original figure 7, unchanged.

7. Figure S1 - the sequence alignment, originally fig. 4, is moved to the supplementary material and increased in size so that it is legible.

8. Figures S2-S13 are unchanged from figures S1-S10 and S13-S14 in the original version, with their order altered to better suit the organization of the Supplementary Material.

9. Figures S-A through S-I now have legible text.

10. Original Figures S11 and S12 were removed because it was not possible to make them legible; these phylogenetic trees will now be provided as Supporting Information.

11. Figures S14 through S17 were added to address concerns and requests from the reviewers, and are addressed in the point-by-point response below.

12. Figure S18 was added as support for statements in the main text regarding AausFP1 nuclear exclusion and aggregation in mammalian cells.

13. Figure S19 was added as support for statements in the main text regarding the absorbance spectra of AausFP2 compared with its C62S mutant.

14. Figure S20 was added as support for statements in the main text regarding alkali denaturation experiments on AausFP2 with and without added β-mercaptoethanol.

New citations related to review

1. Gavrikov AS, Baranov MS, Mishin AS. Live-cell nanoscopy with spontaneous blinking of conventional green fluorescent proteins. Biochem Bioph Res Co. 2019;522(4):852–854. PMID: 31801668. ***Because one peer-reviewed publication has already cited our original preprint, demonstrating that mAvicFP1 displays useful self-blinking behavior at high illumination levels, enabling single-molecule localization microscopy with a single illumination wavelength. Since this manuscript is intended as a Methods and Resources publication, we felt it would be useful to include a reference to a direct application of one of the proteins described here.***

2. Thevenaz P, Ruttimann UE, Unser M. A pyramid approach to subpixel registration based on intensity. Ieee T Image Process. 2020;7(1):27–41. PMID: 18267377. ***This citation is for our use of the StackReg plugin for ImageJ for aligning image stacks from photostability experiments.***

3. Postma M, Goedhart J. PlotsOfData-A web app for visualizing data together with their summaries. Plos Biol. 2019;17(3):e3000202. PMID: 30917112. ***We used this software to generate three of our new figures, displaying all collected data points.***

Point-by-point response to reviewers

Our responses are shown in bold italic font.

Reviewer #1:

The manuscript by Lambert et al. notifies us of nature's profundity. The Aequorea victoria jelly fish species, which provided us with the avGFP, has proven to possess some additional novel fluorescent proteins (FPs). I agree to the last paragraph of the main text in that we now need to explore and understand as much of the molecular biodiversity of glowing creatures.

I found the manuscript somehow disorganized. It was difficult to follow the logic in the Introduction, Results and Discussion. I'd recommend that the manuscript be rewritten more carefully.

While it is difficult to know precisely what aspects of the organization were most problematic to Reviewer #1, we made a strong effort to reorganize the manuscript to make it flow logically as well as possible. We have also asked several colleagues to read over the revised version and have taken constructive feedback into consideration in further revisions to the organization as well.

My comments are as follows.

1) None of supplementary data (figures and tables) are referred to in the main text. Also, the Introduction contains some results; Figures 2 and 3 are already referred to there.

As part of the reorganization of the manuscript, we have moved all results appropriately into the Results and Discussion, shortening the Introduction section. We have rectified the order of figure citations in the main text, and have ensured that all supplementary data are cited in the main text. The order of the supplementary figures and tables was

chosen to best suit the organization of the supplementary material, and so the citations in the main text are not necessarily in numerical order for the supplementary data.

2) Page 2, right, line 5. Please define "AausFP." It should be "A. cf. australis FP."

This correction has been made.

3) Figure 2. What were the concentrations of the protein samples?

Information on protein concentrations was added to the figure legend (now panel B in Figure 1).

4) Page 2, right, lines 19-. The low pKa of AvicFP1, which contains CYG, is explained by analogy to the S65C mutant of avGFP. I think, however, this portion is too speculative.

We agree and have removed this statement from the text, instead noting that the avGFP S65C mutant was originally reported at the same time as S65T.

5) Page 3, left, line 27. I think that doubling time of H2B-mAvicFP1 should be provided.

While this is an unusual request (we could find no papers describing novel FP variants that included H2B fusion doubling times), we felt that this was an experiment worth performing, and so we conceived an experiment that we believe gives the most realistic measure of toxicity - actual long-term live-cell imaging. Supplementary Figure S14 is a plot of doubling times measured for 24 individual cells (daughter cells of 12 originally chosen cells) for transfected and non-transfected cells in the same imaging field, with transfection with H2B-mEGFP or H2B-mAvicFP1. We have added a detailed description of the assay to the Materials & Methods section of the main text and an expanded discussion of the assay and our interpretation of the data in the Supplementary Material. We found that while H2B-mEGFP expression caused U2-OS cells to have a somewhat longer doubling time (cell cycle interval), this was not the case with H2B-mAvicFP1. While we are reluctant to conclude that H2B-mEGFP is toxic or otherwise problematic for cells, we do see these data as an indication that H2B-mAvicFP1 (and mAvicFP1 in general) is unlikely to have any unexpected or unusual toxic effects on cells.

6) Page 3. "It is somewhat ironic that,,," avGFP absorbs principally violet light (400 nm) for the green fluorescence whereas AvicFP1 absorbs blue light. I would rather discuss why avGFP is abundant in A. victoria.

We agree that this statement was not fitting for this manuscript. We have replaced it with some discussion about why avGFP is still the most likely energy acceptor for aequorin, despite the better spectral overlap of AvicFP1 with aequorin emission. Put simply, avGFP is expressed in the same tissue as aequorin (bell margin), while AvicFP1 is not, and so cannot be the FRET acceptor. Any additional discussion about why avGFP is more abundant would be pure speculation, other than to say that it's required for green luminescence emission.

7) Why aren't the two CPs from A. cf. australis called AausCP2 and AausCP3?

We have clarified, in the figure legend for Fig. 2, that FP homologs from each **Aequorea** ***species were given names in order of their discovery, with all proteins receiving the "FP"***

nomenclature for consistency (we could not know prior to characterization which FP homologs were fluorescent proteins vs. chromoproteins).

8) Figure 3. The dotted lines for unconverted spectra are very hard to see.

We have updated this figure (now Fig. 2) to improve visibility of all elements.

9) Figure 4. Hard to see. I would recommend that a relatively simple phylogenic tree be put.

We agree, and have moved the sequence alignment to the Supplementary Material where it can be made larger. We have placed a phylogenetic tree in its place.

10) Page 4, left, lines 10-. Why are the tandem-dimers and monomers hidden?

We have removed statements from the text about tandem-dimers and monomers, as these are appropriately left to future publications. It is far beyond the scope of this manuscript to also include the years-long development of monomeric variants of AausFP1, and we feel that inclusion of this additional element to the manuscript would distract from its main theme - the discovery and thorough characterization of novel **Aequorea** ***FP homologs that can be used as starting material from which to generate monomeric probes for several different imaging modalities.***

11) Page 5, right. About the photoconversion of AvicFP2 and AvicFP3. Could the authors identify the blue-absorption peaks for photoconversion in Figure 1? Also, I am negative on their "rapid" photoconversion, considering that It took seconds to minutes. Ideally, the authors should measure the quantum efficiency of the conversion.

We have added red arrows to Fig. 2 (originally Fig. 3) to clarify the peaks that increase and decrease upon photoconversion for the photoactive proteins in this study. Because measuring the quantum yield of photoconversion accurately proved very difficult for these proteins, especially given the COVID-19 induced lab closure and current low-density campus restrictions, we have instead removed language about the speed/efficiency of conversion and instead improved our qualitative description of the phenomenon we observed for these proteins.

12) There is no information of photostability of the new FPs. To show their practical usefulness, photobleaching data must be presented.

We agree that this is important information for FPs that are intended to be used for probe development. We performed photostability assays in mammalian cells using both widefield and laser scanning confocal microscopy to determine the photobleaching half-life of the two highly fluorescent novel proteins described in this study, AausFP1 and mAvicFP1, compared with the photostability of mEGFP. We found that bleaching rates were very similar prior to correction for molecular brightness, and that both AausFP1 and mAvicFP1 were more photostable than mEGFP after correction for molecular brightness. Detailed methods were added to the Materials & Methods section of the main text, and data are presented in Supplementary Figs. S15 and S16, with extended discussion of the data and methods included in the Supplementary Material.

Reviewer #2:

The work presented in this excellent manuscript involved a collaborative team finding and analysing a new suite of fluorescent proteins from the original Aequorea victoria and a related Aequorea species. The group identified a number of new, interesting and potentially useful fluorescent proteins. Two of these were further characterized by structural biology to provide interesting insights into the molecular basis of function. I guess the most interest going forward is AausFP1 in terms of its overall brightness (which is authors has eluded too), but I am sure there is a lot to be learned (for both fundamental and long-term application) from the others.

We thank the reviewer for these thoughtful comments. We have added information at the end of each subsection of the Results & Discussion section to point the reader toward potential applications for many of the novel proteins described in this manuscript. We feel that the data presented in the revised version of the manuscript are extremely thorough, and we know of no previously published work that has told the full story of FPs from the animal in the wild to applications in cells, with structures and other detailed characterization.

Overall, I thought this was an excellent piece of work with very little to argue with - the science was excellent and I enjoyed reading the manuscript. It is clear that this work is publishable in PLoS Biology as it stands. My reason for minor revisions is that I suggest a couple of changes (and they are minor in the scheme of things).

1. Oligomerisation can play an important role in the spectral properties of fluorescent proteins. As I read it, apart from the engineered mAvicFP1, all the other proteins were oligomers. Do the authors have other data than OSER to confirm the oligomeric status of the proteins (e.g. size exclusion, dynamic light scattering)? Some information is inferred from the two crystal structures but such data can sometimes be a little misleading in terms of oligomeric state in solution. It might also be worth adding a column to Table 1 to state the oligomeric form of each fluorescent protein.

We agree that the OSER assay is only one of many methods to evaluate the monomeric character of an FP. We performed gel filtration and dynamic light scattering analysis of all proteins in this study and now present those data in Fig. S17, along with some discussion in the main text. Importantly, we verified that mAvicFP1 is monomeric, and that avGFP, AausGFP, and AvicFP1 all run as monomers by gel filtration and DLS, indicating that these analysis techniques are not sensitive enough to detect weak dimerization even at fairly high protein concentrations. Because of this, the data from the OSER assay is now an important confirmation that mAvicFP1 is indeed monomeric when expressed in mammalian cells.

We chose not to include oligomerization state in Table 1 primarily because it is unclear for most of the novel proteins. Only a few run as monomers, while the rest run as high molecular weight aggregates or high-order oligomers that we cannot resolve by gel filtration or DLS. We have altered the text to clarify that we cannot currently confirm the oligomeric state of AausFP2 or other proteins to be dimeric, even though the crystal structures (especially the highly homologous dimer interfaces observed in the crystal structures of both AausFP1 and AausFP2) strongly suggest a plausible physiological dimer.

2. Leading on from point 1, it is known that oligomerisation of normally monomeric fluorescent proteins can impact of the spectral characteristics. AausFP1 may be a good example (although given the comments in the manuscript, there is more to be told on this at a later date). A recent good example is DOI: 10.1038/s42004-019-0185-5 concerning how oligomerisation can have a positive impact on GFP properties. It might be good to include such information.

We have added a note about how dimerization can stabilize the chromophore environment, using dTomato as an example. We feel that the publication provided by Reviewer #2 is not completely relevant to this situation because in that case, non-natural amino acids were incorporated at a position known to be critical to the chromophore environment, and therefore is not representative of the behavior of most FPs.

3. Very little is said about maturation times expect that in the methods it appears that certain variants matured at different rates. Could the authors add which proteins matured under which particular conditions.

We added additional detail to the Materials & Methods section about which proteins matured at 37ºC and which required extended maturation on agar plates.

4. While I don't dispute the extinction coefficients measured, I was surprised to see that the proteins where denatured to calculate them. Did the authors look at the values for the native proteins?

We have added additional clarification about the extinction coefficient measurements in the Materials & Methods section. Alkali denaturation has been the standard method of measuring FP extinction coefficients for over 20 years, and is the accepted way of determining this value. The absorbance of the protein is first measured in its native state, and then the protein is denatured with NaOH. The denatured protein then contains a solvent-exposed chromophore with a known extinction coefficient. The absorbance of the denatured protein can therefore be used to calculate the extinction coefficient of the native protein at the same concentration simply by scaling both absorbance spectra to the known extinction coefficient of the denatured chromophore.

Reviewer #3:

This is a very valuable resource to the scientific community in that it identifies a number of new sequences for fluorescent proteins and chromoproteins and characterizes the basic properties of those proteins. These sequences increase the diversity of naturally derived sequences and could be useful for incorporating into efforts aimed at gene shuffling. The paper is clearly written and the resource and results are logically presented. I don't find any flaws with the analysis or presentation. The only thing I would request is that the axes in Figure 3 and the size of Figure 4 be increased because the text isn't legible. Otherwise I think this paper makes a valuable contribution to the scientific community and I support its publication.

We have altered the figures to ensure that all are legible and clear, and thank Reviewer #3 for pointing out these issues.

Reviewer #4:

Lambert G.G. et al. cloned homolog genes of the Aequorea Victoria GFP from Aequorea sp. by mRNA-Seq and de novo transcriptome assembly. Isolated fluorescent proteins (FPs) and chromo proteins (CPs) were characterized, and distinct features such as a chromophore with a crosslink to the main polypeptide chain and reversibly photoswitchability were identified.

To be honest, I got an impression to this manuscript such that the principal claim of the authors is unclear and it seems like a catalog of the FPs and CPs released from the author's group. To discuss the molecular evolution, meticulous comparison with FP/CPs derived from multiple species in the Aequorea sp. is essential. Meanwhile, for claiming the applicability of FP/CPs as a valuable resource for bioimaging, availability to the practical imaging technique using their distinct features must be shown. However, the authors did not seriously address these issues. Therefore, it is difficult to be accepted as a paper worthy to publish from this journal. I recommend for authors to submit this manuscript to much more specific journal.

This manuscript was submitted as a Methods and Resources paper, intended to provide a detailed story of the cloning and detailed characterization of these novel FPs and CPs. We have further characterized the most promising proteins in this set, AausFP1 and mAvicFP1, and shown that one or both have properties that will make them useful for development of novel probes. While we see Reviewer #4's point of view, this paper is intended to provide a Resource of starting materials from which to engineer new imaging probes, rather than to provide a set of ready-made probes. In the past, articles such as this one (for example, Matz. et al 1999 described the cloning of DsRed, Zfp538, and cFP486, among several others, several of which went on to be engineered into bright monomeric probes that are in broad use today). Our group is in the process of engineering bright monomeric probes based on AausFP1, but those efforts are far outside the scope of this article.

To clarify the potential uses of each class of protein we describe in this study, we have added several statements (italicized in the main text) about potential downstream uses of probes engineered from the proteins described here. It is our hope that other groups like ours that specialize in engineering novel imaging probes will develop useful new tools based on the proteins we have discovered.

Minor points:

1. The descRiption in the Introduction section is not match with the contents in the Results and Discussion section.

We have edited the Introduction and Results & Discussion section to remove ambiguity and to ensure that the Introduction does not contain data that belongs in the subsequent section.

2. There are many Supplementary Materials which are not cited in the text.

We have ensured that all Supplementary Materials are cited in the main text.

3. Fig. S12 and Fig. S-A to I in Supplementary Materials are unclear to make out the letters in the figures.

We have replaced Figs. S-A through S-I with figures that contain legible labels, and have moved original Figs. S11 and S12 into their own separate Supplementary Information files so that they are more legible.
