# Supplementary material for: Aequorea’s secrets revealed: New fluorescent proteins with unique properties for bioimaging and biosensing
Source: PLoS Biol. 2020 Nov 2;18(11):e3000936. doi: 10.1371/journal.pbio.3000936 (PMC7660908; doi:10.1371/journal.pbio.3000936)
Supplement: Attachment — Submitted filename: Response to Reviewers PLoS Biology Revision 9-2020.pdf [file pbio.3000936.s008.pdf]

9-29-2020

Point-by-point response to reviewers

Our responses are shown in bold italic font.

Reviewer #1:

I was interested in the observation that AvicFP1 was localized in the body and mouth but not the bell margin where the Aequorin-based bioluminescence occurs.
Page 3, right. Lines 18-
"We can only speculate about what biological imaging might look like today if AvicFP1, rather than avGFP, had been the first FP cloned."
This is a vague sentence. I would suggest that the authors refer to the fact that avGFP was originally discovered as a protein accompanying Aequorin.

We have replaced this sentence (we disagree that it is vague - merely speculative) with the following:

"Originally, avGFP was identified as a partner to the photoprotein aequorin, and this association ultimately led to cloning the cDNA that encodes it. We speculate that other green-emitting FPs were not identified at the same time as avGFP because the brightest visible fluorescence in A. victoria is around the bell margin, while AvicFP1 appears to be expressed exclusively in other tissues (Fig A in S1 Text)."

Page 4, left.
"To our knowledge, there have been no previous reports of chromoproteins with a quantum yield of absolutely zero."
Please specify quantum yield values of AausFP2 and AausFP3 compared to others.

In the interest of time and brevity, we have chosen to remove this sentence rather than reorganize our reference list, since this point is minor and does not affect the conclusions of the paper or the utility of the chromoproteins described (we were unable to measure any fluorescence, as stated in the previous sentence, and so we conclude the quantum yield must be lower than the limit of detection of our most sensitive instruments, essentially zero quantum yield). We are open to adding additional information and/or citations here if the editors feel this is important for the paper.
